# Overlapping toxic effect of long term thallium exposure on white mustard (*Sinapis alba* L.) photosynthetic activity

**DOI:** 10.1186/s12870-016-0883-4

**Published:** 2016-09-02

**Authors:** Radosław Mazur, Monika Sadowska, Łucja Kowalewska, Agnieszka Abratowska, Hazem M. Kalaji, Agnieszka Mostowska, Maciej Garstka, Beata Krasnodębska-Ostręga

**Affiliations:** 1 Department of Metabolic Regulation, Faculty of Biology, University of Warsaw, Miecznikowa 1, 02-096 Warsaw, Poland; 2 Laboratory of Chromatography and Environmental Analysis, Faculty of Chemistry, University of Warsaw, Pasteura 1, 02-093 Warsaw, Poland; 3 Department of Plant Anatomy and Cytology, Faculty of Biology, University of Warsaw, Miecznikowa 1, 02-096 Warsaw, Poland; 4 Laboratory of Ecotoxicology, Institute of Botany, Faculty of Biology, University of Warsaw, Miecznikowa 1, 02-096 Warsaw, Poland; 5 Department of Plant Physiology, Warsaw University of Life Sciences SGGW, Nowoursynowska 159, 02-776 Warsaw, Poland

**Keywords:** Thallium, Heavy metal toxicity, *Sinapis alba* L., Mustard plant, Chlorophyll fluorescence imaging, Photosynthetic complexes, Chemical speciation, Tolerance index

## Abstract

**Background:**

Heavy metal exposure affect plant productivity by interfering, directly and indirectly, with photosynthetic reactions. The toxic effect of heavy metals on photosynthetic reactions has been reported in wide-ranging studies, however there is paucity of data in the literature concerning thallium (Tl) toxicity. Thallium is ubiquitous natural trace element and is considered the most toxic of heavy metals; however, some plant species, such as white mustard (*Sinapis alba* L.) are able to accumulate thallium at very high concentrations. In this study we identified the main sites of the photosynthetic process inhibited either directly or indirectly by thallium, and elucidated possible detoxification mechanisms in *S. alba*.

**Results:**

We studied the toxicity of thallium in white mustard (*S. alba*) growing plants and demonstrated that tolerance of plants to thallium (the root test) decreased with the increasing Tl(I) ions concentration in culture media. The root growth of plants exposed to Tl at 100 μg L^−1^ for 4 weeks was similar to that in control plants, while in plants grown with Tl at 1,000 μg L^−1^ root growth was strongly inhibited. In leaves, toxic effect became gradually visible in response to increasing concentration of Tl (100 − 1,000 μg L^−1^) with discoloration spreading around main vascular bundles of the leaf blade; whereas leaf margins remained green. Subsequent structural analyses using chlorophyll fluorescence, microscopy, and pigment and protein analysis have revealed different effects of varying Tl concentrations on leaf tissue. At lower concentration partial rearrangement of the photosynthetic complexes was observed without significant changes in the chloroplast structure and the pigment and protein levels. At higher concentrations, the decrease of PSI and PSII quantum yields and massive oxidation of pigments was observed in discolored leaf areas, which contained high amount of Tl. Substantial decline of the photosystem core proteins and disorder of the photosynthetic complexes were responsible for disappearance of the chloroplast grana.

**Conclusions:**

Based on the presented results we postulate two phases of thallium toxicity on photosynthesis: the non-destructive phase at early stages of toxicant accumulation and the destructive phase that is restricted to the discolored leaf areas containing high toxicant content. There was no distinct border between the two phases of thallium toxicity in leaves and the degree of toxicity was proportional to the migration rate of the toxicant outside the vascular bundles. The three-fold (nearly linear) increase of Tl(I) concentration was observed in damaged tissue and the damage appears to be associated with the presence of the oxidized form of thallium − Tl(III).

**Electronic supplementary material:**

The online version of this article (doi:10.1186/s12870-016-0883-4) contains supplementary material, which is available to authorized users.

## Background

Light-driven photochemical reactions in higher plants take place in the chlorophyll–protein (CP) complexes localized inside thylakoids, the internal membrane system of chloroplast [1–3]. Core complexes of photosystems I and II (PSI/II) are associated with the respective antennae (LHCI/II) creating the appropriate supercomplexes: LHCII-PSII and LHCI-PSI. The LHCII-PSII and the trimeric complexes of LHCII are localized in stacked regions of the thylakoid membranes (grana), while the LHCI-PSI is exclusively present in unstacked, stroma thylakoids connecting the grana [4–6].

Heavy metals affect plant productivity by interfering with photosynthesis in all aspects of this process and there is a large body of evidence indicating that there is a direct effect of heavy metals on the photochemical reactions. Heavy metals, if present in excessive amount, affect proteins and their functions through binding to histidine, tryptophan and tyrosine residues or disturb the photochemical function of CP complexes by interacting with functional metals and in consequence affecting the photosynthetic electron flow [7–10]. Surplus of heavy metals in the cells leads to damages of the photosynthetic pigments due to a substitution of their central atoms. For example the Hg ions can substitute Cu ions in plastocyanin, while Cu or Cd ions can replace Mg in the chlorophyll molecules. Inhibition of enzymatic reactions can also be caused by substitution of enzyme cofactors by heavy metal ions. The Cd ions can substitute Ca^2+^ in the Oxygen Evolving Complex (OEC) and can interact with the non-heme Fe ions on the acceptor side of the PSII. Furthermore, the loss of the extrinsic proteins of OEC is a result of the direct action of Cu(II) [11]. Cu, Cd, Pb and Zn ions affect the photosynthetic efficiency indirectly by inhibiting enzymes responsible for the chlorophyll synthesis, RuBisCo and other Calvin cycle enzymes [8, 11]. Qualitative composition and quantitative content of carotenoids, both essential for the photochemical functions of CP complexes and the stability of thylakoid membranes, were influenced by an excess of Cu and Cd ions [8, 11–13]. Moreover, the indirect effect of heavy metals was observed on the chloroplast proteome, with varying protein composition and stability of CP complexes [9, 14]. In mustard plants the excess of Cd ions was reported to cause a marked decrease in the LHCII and ATP-ase subunit levels [15], while in rye a decrease in the molecular mass of Lhcb1 and Lhcb2 proteins was observed [13]. Cd and Cu ions had opposite effect (a decrease by Cd and an increase by Cu) on the LHCII aggregation due to changes in the protein and xanthophyll composition of complexes, respectively [13]. These opposite effects of the Cd and Cu toxicants suggest a complex and intricate inhibitory mechanism of heavy metal action. So far, there is very limited data on direct and indirect action of thallium on the photosynthetic apparatus in higher plants.

The influence of heavy metals on the ultrastructure of cells has been also intensively investigated. Distortion of the chloroplast structure by heavy metals was manifested by changes in size and stacking of grana, by thylakoid swelling, and also by the plastoglobule and starch accumulation [8]. Disintegration of the thylakoid membranes may be partially related to an enhanced lipoxygenase activity and a reduction of the galactolipid level [8]. Furthermore, the heavy metals, by inducing generation of the reactive oxygen species, influenced the lipid peroxidation [7], which might cause the disturbance of thylakoid structure [11]. Additionally, the quantitative and qualitative changes in composition of CP complexes [13] might result in a noticeable disorder of the thylakoid structure.

Thallium (Tl) is a natural trace element, widely distributed in Earth's crust, found in small amounts in sulfide (Fe, Zn, Cu, Pb) and selenite ores (Cu, Ag). Major anthropogenic sources of thallium pollution are mining, flotation treatment and smelting and areas of relatively high content of thallium are found all over the world, for example in Poland [16], Italy [17, 18], Spain [19], Turkey [20], Chile [21] and China [22]. Thallium is considered to be one of the most toxic of heavy metals [23]. It does not have a known biological function and seems not to be an essential element for life. Thallium is a chemical analogue of potassium and the mechanism of its toxicity as Tl^+^ is that it can substitute K^+^ due to a similar ionic radii of both ions [24].

Elevated concentrations of Tl were found in tissues of organisms living in the areas polluted with the toxicant [25, 26]. Some of the plants, especially species of the *Brassicaceae* family are able to hyperaccumulate Tl ions and the accumulation ranged from up to 1,489 mg Tl kg^-1^ DW in shoots of *Silene latifolia* [27] and 2,810 mg Tl kg^-1^ DW in *Iberis intermedia* [28–30], to 15,200 mg Tl kg^-1^ DW in *Biscutella laevigata* [17, 31, 32]. High ability of crop plants such as *Armoracia rusticana* [33], *Sinapis alba* [34, 35], and *Brassica* sp. [36, 37] to accumulate Tl has been demonstrated and threshold concentration of Tl hyperaccumulation in shoots was recently established at 100 mg Tl kg^-1^ DW [38]. However, the investigated crop species accumulated Tl under laboratory conditions and none of them colonized Tl-enriched soils and they cannot be considered as Tl hyperaccumulators. High accumulation of thallium was also observed in *Iberis intermedia* grown in soil spiked with TlCl solution [39].

Elevated concentration of thallium induced oxidative stress resulting in damage to lipids and proteins [40–42], but the mechanism of the toxicant action is far from being elucidated. In contrast to wide-ranging studies of the effect of other heavy metals toxicity on photosynthetic reactions [9], there is paucity of data in the literature concerning thallium effects in higher plants. Aoki *et al.* [41] investigated Tl-induced changes in unicellular freshwater cyanobacteria (*Synechocystis* sp*.*) – an accepted model organism in studies on the photosynthesis. In this organism the integrity of the photosynthetic apparatus was affected by Tl exposure with observable fragmentation of thylakoid membranes and accumulation of Tl in cells. These changes were accompanied by markedly decreased photosynthetic activity of cells [41].

The aim of this investigation was to identify the main sites of the photosynthetic process in leaves of white mustard, which may be directly or indirectly inhibited by Tl. The ultrastructure, pigment and protein composition comparisons and low-temperature fluorescence analyses in Tl affected plants enabled identification of quantitative and qualitative re-arrangements in the thylakoids and CP complexes in response to the toxicant. The functionality of PSII and PSI as well as layout of PSII inhibition were assayed by measuring the photosystem photochemistry using a modulated fluorescence or absorbance and fluorescence imaging on the leaf surface. Relations between structural and functional data as well as probable mechanisms of thallium action, such as the importance of the Tl(I) and Tl(III) ions are discussed in detail.

## Methods

### Plant growth conditions

Plant cultivation was carried out in a growth chamber at 22 °C/ 20 °C (day/night) with 16 h photoperiod at 100 μmol of photons m^-2^s^-1^ of photosynthetically active radiation (PAR). White mustard (*Sinapis alba* L. *cv*. Maryna) seeds (from Agro-land S.C., Wróblewskiego 19 St., 93-578 Łódź, Poland) were sown in an artificial medium (glass balls) and seedlings at cotyledon stage were placed in 11 L pots (24 plants per pot) filled with aerated liquid nutrient solution containing: 3 mM Ca(NO_3_)_2_, 1.5 mM KNO_3_, 1.2 mM MgSO_4_, 1.1 mM KH_2_PO_4_, 0.1 mM C_10_H_12_N_2_O_8_FeNa, 5 μM CuSO_4_, 2 μM MnSO_4_ × 5 H_2_O, 2 μM ZnSO_4_ × 7 H_2_O, and 15 nM (NH_4_)_6_Mo_7_O_24_ × 4 H_2_O, pH 6.0-6.5. Thallium (in a form of TlNO_3_), in amounts of 0 (control cultivation), 100, 500 and 1,000 μg L^−1^, was added to the nutrient solution. Nutrient solution was refilled, if necessary, without additional dose of thallium during the cultivation. After 4 weeks the plants were harvested. This time period corresponded to full vegetative growth of control plants just before appearance of flower buds. The healthy leaves from plants grown in the presence of 100 μg L^−1^ Tl were denoted as H100. The leaves of plants cultivated in the presence of 500 μg L^−1^ Tl were divided into three groups: (*i*) healthy green leaves, which showed no changes in morphology in comparison with that of leaves of the control cultivation (H500); (*ii*) green parts of the affected leaves (G500) and (*iii*) yellow parts of the affected leaves (Y500). Similar classification was applied to the leaves from plants grown in the presence of 1,000 μg L^−1^ Tl (G1000 and Y1000).

### Determination of the root tolerance index

The degree of tolerance to Tl was determined using the root tolerance index [43, 44]. The length of the root of each plant was measured with a millimeter ruler. The measurements started before thallium salt was added to the medium and were repeated every three days until the end of the cultivation (i.e., to the harvesting). On the basis of measurements, the tolerance index (IT) (the growth of roots of plants exposed to thallium, expressed as a percentage of control roots growth) was calculated.

The concentration of Tl in mineral medium was monitored regularly during the experiment. A volume of 10 mL of medium was taken from each container and analyzed by the ICP MS technique as described below. Initial concentration of Tl was measured immediately after Tl salt was added to the containers and the measurement was repeated weekly through the cultivation.

### Microscopic observations

Samples of 3 mm^2^ were cut from middle parts of the leaves. These samples were fixed in 2.5 % (w/v) glutaraldehyde in 50 mM cacodylate buffer (pH 7.4) for 2 h, washed with the fixing buffer and placed in a 2 % (w/v) OsO_4_ in 50 mM cacodylate buffer (pH 7.4) for approximately 12 h at 4 °C. Specimens dehydrated in a graded acetone series, were embedded in a low viscosity epoxy resin and cut on a Leica UCT ultramicrotome. Semi-thin sections of 250 nm thick and ultrathin sections of 70 nm were used for light and transmission electron microscopy (TEM), respectively. For TEM technique the ultrathin sections were stained with uranyl acetate and examined with a JEM 1400 electron microscope (Jeol, Japan) of the Nencki Institute of Experimental Biology of the Polish Academy of Sciences, Warsaw. For bright field light microscopy the semi-thin sections were stained in 0.5 % (w/v) toluidine blue water solution and examined with a Zeiss AxioLab A1 microscope equipped with Zeiss A-Plan 20 × (NA = 0.65) Ph2 objective lens.

### Chlorophyll *a* fluorescence imaging

Leaf fluorescence images were recorded with FluorCam FC 800-C (Photon System Instruments, Brno, Czech Republic). Leaves from dark adapted plants for 30 min were detached immediately prior placing them on tray in a FluorCam chamber. The fluorescence images were recorded with 512 × 512 pixels resolution with camera parameters set to avoid saturation of CCD wells. After F_0_ and F_M_ determination (0.8 s saturation pulse with 4,000 μmol photons m^−2^ s^−1^) and 30 s dark relaxation the actinic light (50 μmol photons m^−2^ s^−1^) was on and five saturation pulses at constant time intervals were applied. After 120 s actinic light was turned off and one additional saturation pulse, during 20 s dark relaxation, was applied. At all saturation pulses the F_M_’ values were measured. The recorded data were analyzed with FluorCam v7.0 software. The maximal quantum efficiency of PSII (F_V_/F_M_) was calculated from the formula (F_0_ – F_M_) / F_M_ and the non-photochemical quenching parameter (NPQ) from the formula: (F_M_ – F_M_’) / F_M_’.

### Simultaneous chlorophyll *a* fluorescence and P700 measurements

Fluorescence measurements were carried out using a pulse-amplitude modulation fluorometer, a Dual-PAM-100 (Heinz Walz GmbH, Effeltrich, Germany) and the automated Induction Curve routine provided by the DualPam software, with repetitive application of saturation pulses for assessment of fluorescence and P700 curves, from which the specific parameters of photosystem I (PSI) and photosystem II (PSII) were calculated by the software. Plants were dark adapted for 30 min prior to all measurements. After F_0_ (red light pulse with intensity below 1 μmol photons m^−2^ s^−1^), F_M_ (0.3 s red light saturation pulse with 10,000 μmol photons m^−2^ s^−1^) and P_M_ (0.3 s red light saturation pulse with 10,000 μmol photons m^−2^ s^−1^ after 10 s far red light illumination) determination the actinic light (50 μmol photons m^−2^ s^−1^) was on and F_M_’ and P_M_’ values were measured by application of series of saturation pulses of identical light intensity (0.3 s pulse at 10,000 μmol photons m^−2^ s^−1^ with 1 s interval between actinic light on and saturation pulse, and with 20 s interval between two consecutive saturation pulses).

The effective PSII quantum yield (Y(II)) was calculated according to Genty *et al.* [45] by the formula: Y(II) = (F_M_′ − F) / F_M_′ and measured after dark adaptation when the effective PSII quantum yield is maximal. The value of Y(II) varies between 0 and 1. Kramer *et al.* [46] introduced two new parameters describing non-photochemical processes in PSII: Y(NPQ) and Y(NO). The first parameter is the yield of regulated energy dissipation in PSII and is related to the xanthophyll cycle activity, whereas the second one, Y(NO) represents the yield of nonregulated energy dissipation in PSII and accordingly represents the effect of side reactions after prolonged closure of the PSII centers. By definition the sum of all yields always gives unity: Y(II) + Y(NO) + Y(NPQ) = 1. The photochemical quantum yield of PSI (Y(I)) is described as the fraction of overall P700 that in a given state is reduced and is not limited by the acceptor side. The Y(I) value was calculated from complementary non-photochemical PSI quantum yields: Y(I) = 1 − Y(ND) − Y(NA), where Y(ND) is non-photochemical energy dissipation related to donor side limitation and Y(NA) is non-photochemical energy dissipation related to acceptor side limitation [47].

### 77 K steady-state fluorescence measurements

Leaf samples were ground in a mortar in chilled 20 mM HEPES-NaOH buffer (pH 7.5), containing 330 mM sorbitol, 15 mM NaCl and 4 mM MgCl_2_. Homogenates were filtered through 100 μm nylon mesh, diluted to chlorophyll concentration below 10 μg mL^-1^ and immediately subjected to the fluorescence measurements. Low temperature (77 K) fluorescence emission spectra were recorded using modified Shimadzu RF-5301PC spectro-fluorometer where excitation and emission beams were supplied by optical fibers. Samples were placed in a polytetrafluoroethylene cuvette and submerged in liquid nitrogen. Excitation wavelength was set at 470 nm, excitation and emission slits at 5 nm and scans were taken from 600 to 800 nm through LP600 filter. Due to a decrease in the fluorescence caused by the chlorophyll reabsorption effect, the measured fluorescence was compensated by multiplication by a correction factor: antilog_10_(A_ex_/2 + A_em_/2), where A_ex_ and A_em_ are sample absorption at the excitation and emission wavelength respectively [48].

### Pigments extraction and analysis

Chlorophyll concentration was measured directly in leaf blade using SPAD-502 chlorophyll meter (Konica Minolta, Japan) as well as by spectroscopic measurements following extraction with 80 % acetone [49]. For further analysis of carotenoids and chlorophylls by UPLC MS, a multi-step extraction procedure was applied as described previously [50].

Extracted pigments were separated using Acquity Ultra Performance LC system (Waters) connected with Synapt G2 HDMS mass spectrometer (Waters). The samples were injected (7.5 μL) into an Acquity UPLC HSS T3 (1.8 μm, 1.0 × 150 mm) analytical column equilibrated in 100 % of solvent A (water/methanol, 15/85; v/v). The column was washed for 100 min at a constant flow rate of 35 μL min^−1^ with 100 % of solvent A at 25 °C. Samples were fractionated with the step gradient of buffer B (methanol/2-propanol/hexane, 2/1/1; v/v) in buffer A as follows: 0-15 % B in the 100-160 min (flow rate 35 μL min^−1^); 15-80 % B in 160-240 min (flow rate 35-20 μL min^−1^); 80-90 % B in 240-245 min (flow rate 20 μL min^−1^); 90-100 % B in 245-250 min (flow rate 20-80 μL min^−1^) and hold for 10 min at 100 % B. In the next 5 min concentration of solvent B was decreased to 0 % and the column was equilibrated for 10 min with 100 % buffer A (flow rate 80 μL min^−1^) before the next injection. Fractionation of samples was monitored by a photodiode array detector at 200-750 nm range and mass spectrometer at 200-1000 m/z range. For better ionization efficiency the column eluate was mixed with LiOH solution (50 mM in methanol/water, 39/1; v/v) distributed by an external syringe pump (flow rate 2 μL min^−1^). The mass accuracy of the raw data was corrected using leucine enkephalin standard [51] that was used as a lock mass during sample analysis. For quantification the single chromatogram at 436 nm (characteristic for carotenoids) was extracted and integrated using MassLynx 4.1 software (Waters).

### SDS-PAGE and Western-blot analysis

For electrophoretic analysis leaf extracts were prepared with Laemmli Sample Buffer (Bio-Rad Laboratories, USA) and samples containing 0.25 μg of chlorophyll were separated on a 14 % (w/v) polyacrylamide gels. SDS-PAGE and Western-blot analysis were performed as described by Janik *et al.* [52]. Briefly, following separation by SDS-PAGE and electrotransfer onto the polyvinylidene fluoride (PVDF) membrane, the specific polypeptides were detected with primary antibodies (all raised in rabbits, from Agrisera, Sweden): Lhcb1 (AS01 004), Lhcb2 (AS01 003), D1 (AS10 704), D2 (AS06 146), CP43 (AS11 1787), PsbO (AS05 092), Lhca1 (AS01 005), PsaA (AS06 172) followed by anti-rabbit horseradish peroxidase conjugate and ECL Detection System (Bio-Rad Laboratories, USA). Immediately after detection the membrane was stripped by incubation (2 × 15 min, 55 °C with shaking) in 62.5 mM Tris-HCl (pH 6.8) buffer containing 2 % (w/v) SDS and 100 mM 2-mercaptoethanol and washed in 20 mM Tris-HCl buffer (pH 7.5), containing 0.5 M NaCl and 0.1 % (v/v) Tween 20. Subsequently the reaction with the new set of antibodies with the same detection system was performed.

### Determination of total thallium concentration

The leaves were air dried (55-60 °C) and homogenized using agate ball mill. The samples were digested in nitric acid (200 mg in 3 mL conc. HNO_3_). The mineralization was carried out in a closed system with automatic temperature control (Ethos 1600 Milestone, Italy). Microwave energy of 1,000 W was applied. The temperature control settings were as follows: 5 min, 90 °C; 10 min, 170 °C; 45 min, 200 °C. Following digestion the samples were diluted with water to the total volume of 25 mL and analyzed by ELAN 6100 DRC ICP mass spectrometer (PE-SCIEX, Canada). Nutrient solutions were diluted 10 or 100 fold with water and also analyzed by ICP MS. Measurements were performed applying the following experimental parameters: sweep: 5; number of replicates: 5; dwell time: 0.1 s; ICP RF power: 1,100 W; lens voltage: 12 V; nebulizer gas flow: 0.95 L min^−1^; plasma gas flow: 13.3 L min^−1^; and monitored isotopes: ^203^Tl and ^205^Tl. Quantitative Analysis program was used to automatically correct the intensities of interfering isobaric and molecular ions. For quantitative determinations the calibration curve method was applied. The signal intensity drift was controlled by determination of a standard solution after analysis of 5-10 samples.

### Thallium speciation analysis

Selected leaf tissues were ground to a fine powder in liquid nitrogen and extracted with 100 mM ammonium acetate, 5 mM diethylenetriaminepentaacetic acid (DTPA) solution pH 6.2; 1 g of plant material in 8 mL of extraction solution was shaken for 1 h at 37 °C. The extracts were clarified by centrifugation at 21,000 *g* for 30 min and the supernatant was diluted 10fold with the extraction solution. Nutrient solutions were diluted 10 fold with the extraction solution to conserve thallium speciation. Thallium species were separated on a size exclusion column Superdex Peptide 10/300 GL (13 μm, 10 × 310 mm), using 1200 HPLC pump (Agilent) with injection valve model 7725 and a 100 μL injection loop (Rheodyne, Cotati). Mobile phase was 100 mM ammonium acetate with 5 mM DTPA, pH 6.2, at a flow rate of 0.75 mL min^−1^. ^203^Tl and ^205^Tl were detected on-line by ELAN 6100 DRC ICP mass spectrometer (PE-SCIEX, Canada) with a concentric nebulizer. Measurements were performed with the following experimental parameters: nebulizer gas flow 0.84 L min^−1^, auxiliary gas flow 1.2 L min^−1^, plasma gas flow 14.5 L min^−1^, ICP RF power 1,000 W, sweeps/reading 1, readings/replicate 14,000, replicates 1, dwell time 100 ms.

### Analysis of the results

For determination of the statistical significance of the differences between groups two types of statistical analysis were performed. For root tolerance index data (*n* = 24) the ANOVA analysis with post-hoc Tukey test at *p* = 0.05 was used. For the rest of the data where the number of replicates between the groups was different (n between 3 and 10, details in figure legends) the non-parametric Kruskall-Wallis analysis with post-hoc Mann-Whitney test at *p* = 0.05 was employed. All analyses were carried out using Statistica 9.1 software package (Statsoft Inc., USA).

## Results

### Tolerance to thallium

**Fig. 1:**
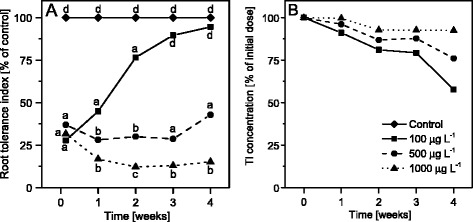
Tolerance of *S. alba* to different concentrations of Tl. **a** Tolerance index (IT) of *S. alba* to Tl during 4 weeks of cultivation. Mean values of IT among groups (control group is 100 % every time) denoted by the same letter did not differ significantly at *p* = 0.05 (*n* = 24, for clear picture the SD values were omitted). **b** total Tl concentration in mineral medium during 4 weeks of cultivation expressed as a % of an initial dose and measured by ICP MS

The root tolerance test revealed that exposure of plants to each of the applied concentrations of Tl resulted in clearly different effects. These differences were already noticeable after the first week of the exposure and became more pronounced during the course of the experiment (Fig. 1a). At 100 μg L^−1^ exposure, growth of roots was strongly inhibited only at the beginning of cultivation (IT value was on average 28 % of control) and after the third week of experiment root growth rate did not differ from that of the control (Fig. 1a), and the amount of Tl in the medium decreased by a half during the course of the experiments (four weeks; Fig. 1b). These observations indicated that *S. alba* plants taken up Tl from the medium and tolerated well the exposure to the toxicant at 100 μg L^−1^. The root tolerance index at the concentration of 500 μg L^−1^ was at the level of 30-45 % of control but it increased during the last week of cultivation. The difference in IT index at 500 μg L^−1^ was statistically significant in comparison with IT indices for 100 and 1000 μg L^−1^ exposures (Fig. 1a). At 500 μg L^−1^ exposure the plants taken up approximately 25 % of Tl from the medium and exhibited moderate toxicity. The initial concentration of 500 μg L^−1^ of Tl was reduced by 25 % during four weeks (Fig. 1b). At the concentration of 1000 μg L^−1^ of Tl, the growth of roots was strongly inhibited during the whole time of experiment and the IT index decreased to 12-15 % of control, indicating strong toxicity to *S. alba* (Fig. 1a). In this case the decrease of Tl concentration in the medium was the smallest (Fig. 1b).

### Changes in leaf morphology under thallium exposure

White mustard plants grown in the presence of Tl in a hydroponic solution exhibited many morphological defects (Additional file 1: Figure S1). However, these defects were visible only at higher concentrations of Tl, while plants exposed to 100 μg L^−1^ of Tl were not morphologically different from the control plants (Additional file 1: Figure S1A, B). The plants exposed to 500 μg L^−1^ Tl were much smaller and a half of them showed changes in the leaf blade morphology with discoloration along the midribs and the main nerves, and deformation of the margins (Additional file 1: Figure S1C). At 1000 μg L^−1^ the plant growth was highly inhibited – plants developed only six strongly deformed leaves; control plants had nine leaves. Progressive discoloration areas and necrotic regions were visible on the surface of the older leaves (Additional file 1: Figure S1D). Moreover, approximately 15 % of seedlings did not survive exposure to Tl at 1,000 μg L^−1^.

### Total concentration and chemical speciation of thallium

**Fig. 2:**
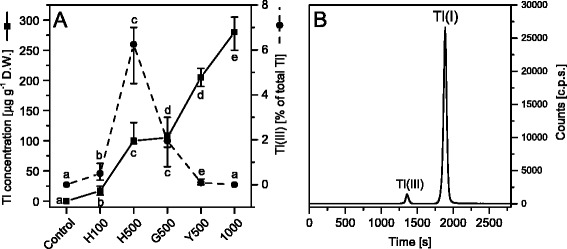
Analysis of thallium accumulation and speciation in *S. alba* leaves. **a** Total Tl concentration (solid line with full squares) and the contribution of Tl(III), expressed as a % of total Tl content, (dashed line with full circles) in leaves of white mustard grown in control and 100 (H100), 500 (H500, healthy green leaves; G500 and Y500, green and yellow parts of the affected leaves) and 1,000 μg L^−1^ of Tl. Values are medians from three to four replicates and whiskers show the range of the data. Values denoted by the same letter did not differ significantly at *p* = 0.05. **b** An example of SEC ICP MS chromatogram of white mustard leaf extract (H500). Mobile phase: 100 mM ammonium acetate with 5 mM DTPA, pH 6.2; flow rate 0.75 mL min^−1^; detected isotope: ^205^Tl

The total concentration of Tl in leaves increased with the increase of Tl concentration in the nutrient solution (Fig. 2a). For plants grown in the presence of 500 μg L^−1^ Tl, the concentration of Tl in healthy leaves (H500) and in green parts of the affected leaves (G500) was similar. The yellow parts of leaves (Y500) accumulated almost twice as much Tl as the green parts (H500 and G500).

Thallium is present in the environment in two redox states, namely Tl(I) and Tl(III). Chemical speciation analysis of Tl in leaf extracts was performed using HPLC with a size exclusion column (SEC). An example of this analysis is presented in Fig. 2b where the first signal corresponds to Tl(III), detected as a higher mass complex with DTPA and the second signal for free ions of Tl(I). In each sample the dominating ion form was Tl(I), but noticeable amounts of Tl(III) were also detected: 0.5 % of total Tl concentration in H100, 6 % in H500, 1.9 % in G500 and 0.1 % in Y500. In leaves grown in the presence of 1,000 μg L^−1^ Tl only trace amounts (not sufficient for quantitative determination) of Tl(III) were found (Fig. 2a).

Additionally, speciation of Tl was determined in nutrient solutions, which originally contained Tl(I) only. Samples of nutrient solutions were collected at the same time as the plants were harvested. Samples were diluted (1:9) with a mobile phase solution in order to conserve chemical speciation of Tl. Tl(III) was not detected in samples prepared in this way - after several weeks of the plant cultivation all nutrient solutions still contained only Tl(I).

### Changes in leaf blade anatomy

Additional analysis of the leaf blade anatomy by light and electron microscopy was also performed in attempt to explain morphological changes and discoloration of leaves on plants grown in the presence of 500 and 1,000 μg L^−1^ Tl.

**Fig. 3:**
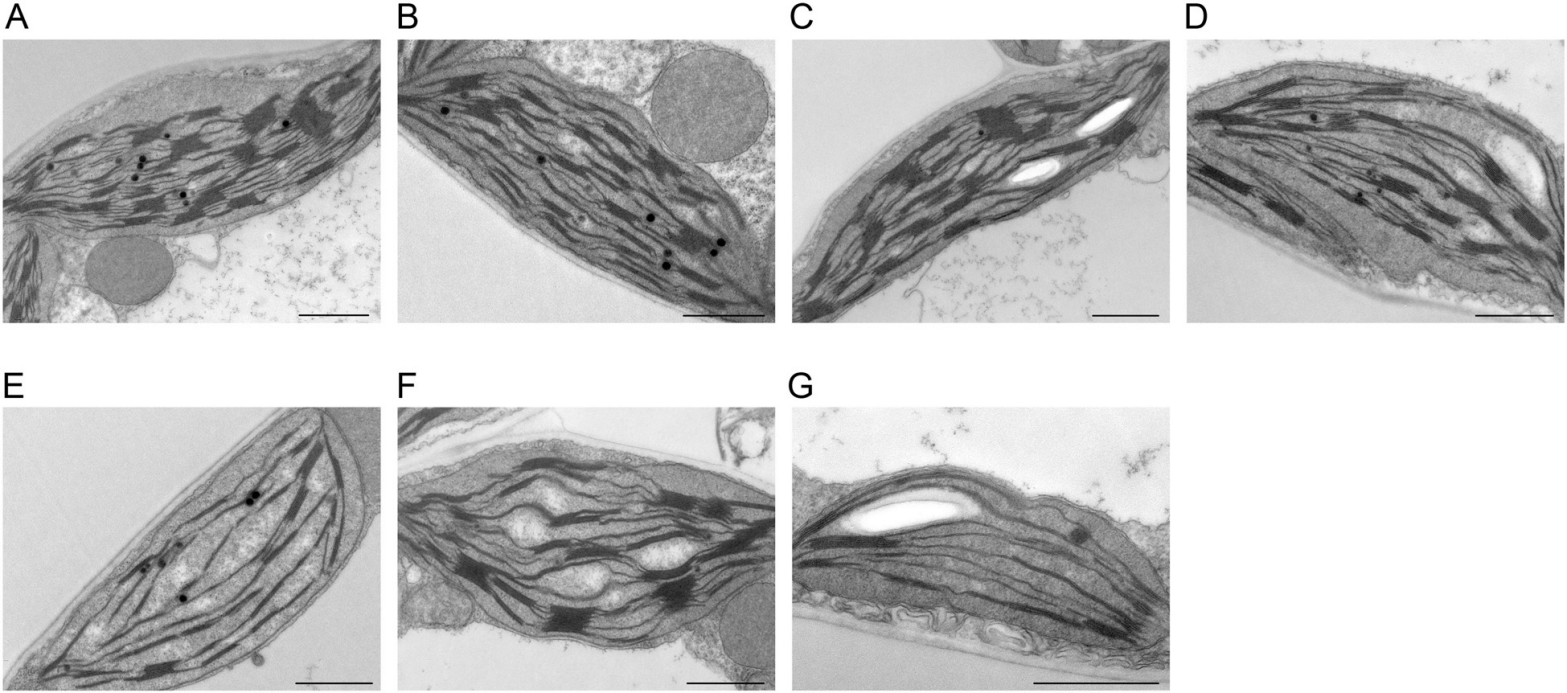
Changes of mesophyll chloroplast structure of *S. alba* plants grown in the presence of thallium. TEM images of chloroplasts from control (**a**) and Tl-treated plants (**b**-**g**): 100 μg L^−1^ (H100) (B); 500 μg L^−1^ healthy green leaves, (H500) (**c**), green (G500) (**d**) and yellow (Y500) (**e**) parts of the affected leaves; 1,000 μg L^−1^ green (G1000) (**f**) and yellow (Y1000) (**g**) parts of the affected leaves. Bar = 1 μm

Analysis of the leaf blade cross-sections (Additional file 1: Figure S2) showed no significant changes in the cell structure between control and the plants exposed to Tl. However, in leaves of the Y500, G1000 and Y1000 samples there was a higher number of the palisade mesophyll cells, which in these samples formed two quite regular layers whereas in control and in the H100 leaves these cells were irregularly arranged. Higher number of the palisade mesophyll cells resulted in an overall increase of the leaf blade thickness. Detailed analysis of the cell structure by a transmission electron microscopy showed no presence of high electron density deposits usually related to complexes containing heavy metals [53]. No anomalies in structural appearance of nuclei, mitochondria, peroxisomes and endoplasmic reticulum were detected in Tl treated plants. However, changes in the chloroplast structure emerged with the exposure to increased Tl concentrations in the nutrient solution (Fig. 3). Chloroplasts in the control plants had a typical structure – thylakoid network was well developed with high number of grana stacks, composed of 10-15 layers on average and connected by unstacked stroma thylakoids (Fig. 3a). Exposure to low concentration of Tl (100 μg L^−1^) did not affect the thylakoid structure (Fig. 3b) and similarly chloroplasts in H500 and G500 leaf samples showed no changes in the thylakoid membrane organization (Fig. 3c, d). However, in the Y500 samples the chloroplast size was smaller (Fig. 3e) and there was a significant decrease of the thylakoid membrane length as well as dramatic reduction of grana stacks. Chloroplasts in G1000 samples showed no significant changes in the thylakoid structure compared to the control plants (Fig. 3f). However, chloroplasts in the Y1000 samples were much smaller than those present in the control samples. Similar to Y500 in these plants the total length of thylakoid membranes and the number of grana were very low (Fig. 3g) as compared to the control chloroplasts.

### Changes in photochemical activity - in vivo fluorescence measurements

**Fig. 4:**
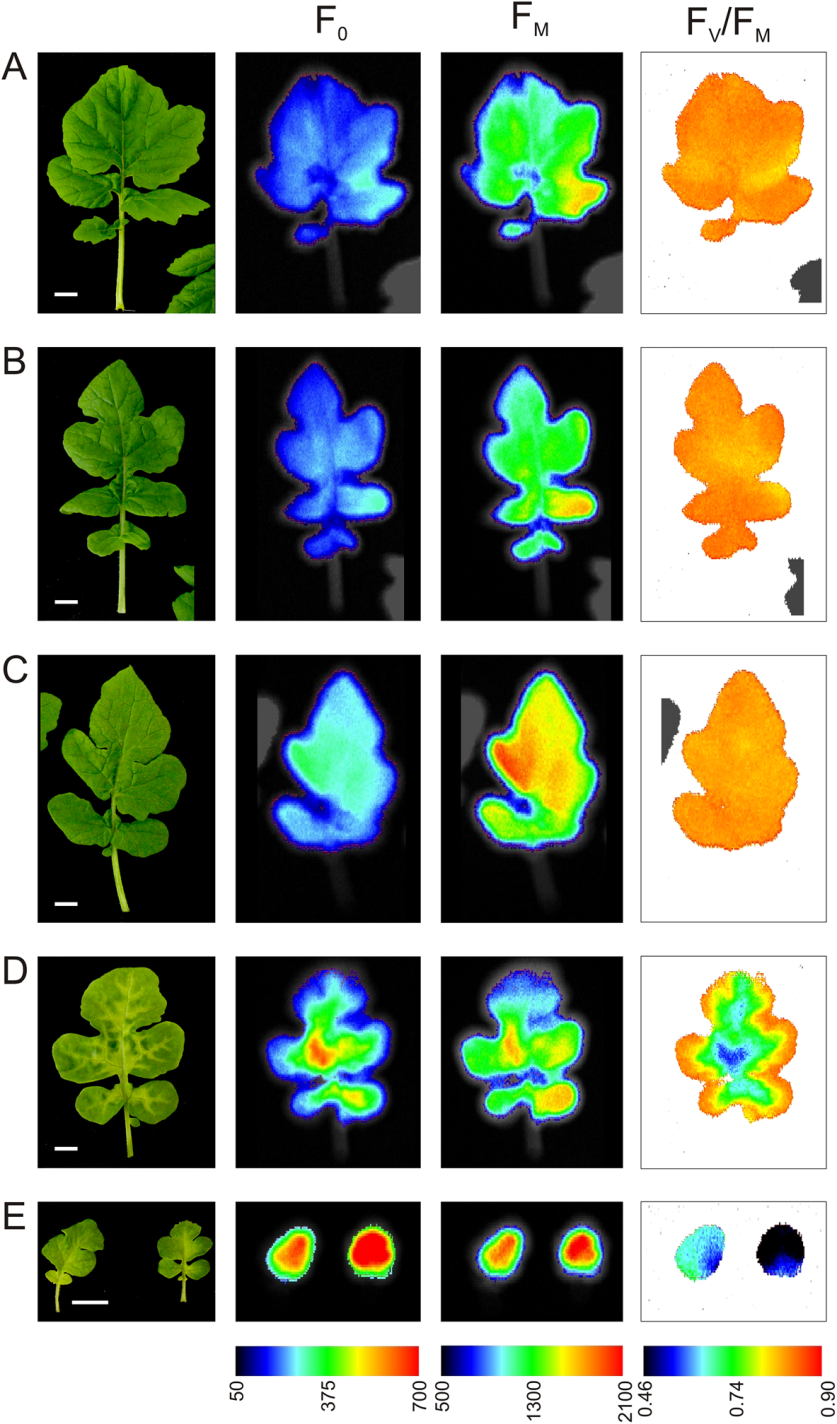
Chlorophyll *a* fluorescence imaging of *S. alba* leaves from control and Tl-treated plants. Pictures present (from left to right): leaf morphology, minimal (F_0_) and maximal (F_M_) chlorophyll fluorescence distribution and calculated F_V_/F_M_ value of control (**a**) and Tl-treated plants (**b**-**e**): 100 μg L^−1^ (**b**); 500 μg L^−1^ healthy (**c**), and affected (**d**) leaves; 1,000 μg L^−1^ (**e**). The images are representative for at least ten leaves from each treatment. Bar = 1 cm

The distribution of chlorophyll fluorescence in the dark adapted plants was analyzed to ascertain how these relate to changes in the chloroplast structure observed at elevated levels of Tl (Fig. 4). In control leaves (Fig. 4a) the F_0_ intensity was almost uniformly distributed throughout the leaf surface and its average intensity was 197 ± 21 fluorescence units. Application of the saturation pulse induced a 6-fold increase in the signal and it reached the maximal fluorescence (F_M_) level. The calculated maximal quantum efficiency of PSII (F_V_/F_M_) for the entire leaf had a Gaussian distribution with the maximum at 0.83, which is a typical value for plants grown in optimal conditions [54]. In H100 leaves the fluorescence distribution as well as F_V_/F_M_ value were the same as in the control leaves (Fig. 4b). Elevated concentration of Tl in leaves influenced both F_0_ and F_M_ in H500 leaves: the value of F_0_ increased in the leaf center, however F_M_ increased as well and as a result the F_V_/F_M_ was the same as in the control plants (Fig. 4c).

In the morphologically affected leaves from plants grown in the presence of 500 μg L^−1^ Tl the value of F_0_ increased gradually from the edge to the center of the leaf blade (Fig. 4d) and reached the level similar to that in the discolored areas of the leaves. Increased intensity of F_0_ strongly influenced the F_V_/F_M_ value, which had optimal values only at the leaf margin and decreased gradually to 0.70-0.62 in the center (Fig. 4d). In plants exposed to 1,000 μg L^−1^ of Tl the F_0_ signal was very high (three times more intense than in the controls), especially in the center of the leaf (Fig. 4e) and as a result the observed average F_V_/F_M_ values were very low, between 0.30 and 0.67 depending on the analyzed leaf (Fig. 4e).

**Fig. 5:**
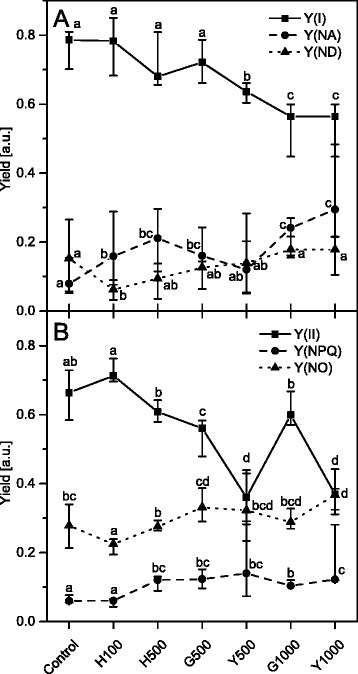
PSI and PSII activity of *S. alba* leaves from control and Tl-treated plants. The photochemical and non-photochemical yields of PSI (**a**) and PSII (**b**) were measured in the steady state light conditions. Y(I) and Y(II) are photochemical yields of PSI and PSII respectively. Y(NA) and Y(YD) are the non-photochemical yields of PSI related to acceptor and donor side limitation respectively. Y(NPQ) and Y(NO) are non-photochemical yields of regulated and non-regulated energy dissipation processes in PSII respectively. Values are medians from three to ten replicates and whiskers show the range of the data. Values denoted by the same letter did not differ significantly at *p* = 0.05. Description of samples abbreviation as given in the legend to Fig. 3

The decrease of the F_V_/F_M_ value indicated that the photochemical activity in the Tl exposed plants was impaired, especially in the discolored areas of the leaves. The photochemical and non-photochemical activity yields in steady state conditions (calculated after the last saturation pulse during the induction curve routine) for PSI and for PSII are presented in Fig. 5a and b, respectively. The photochemical activity of PSI (Y(I)) and PSII (Y(II)) in control plants showed typical values observed in other species [55, 56]. With increasing concentration of Tl the Y(I) and Y(II) values decreased and reached the minimal values in the Y1000 plant samples (Fig. 5a, b).

The decreasing value of Y(I) indicated that the excitation energy reaching PSI centers is more effectively converted to heat by non-photochemical process. With increasing Tl concentration in leaves fluctuations were observed in Y(ND) and Y(NA) yields, however, the rising trend for both non-photochemical yields was observed and these reached their maximal values in the Y1000 samples (Fig. 5a).

The decrease of Y(II) yield, similar to Y(I) (Fig. 5b), indicated that the photochemical conversion of the excitation energy that reaches PSII centers was decreasing suggesting that a non-photochemical conversion was more efficient*.* With the increase of Tl concentration in leaves the Y(NPQ) and Y(NO) values increased together with some fluctuations similar to those for the PSI yields. In Y1000 samples the Y(NPQ) yield increased 2-fold whereas the increase of Y(NO) yield was only 20 % higher than that of the controls (Fig. 5b).

The observed increase of PSII non-photochemical yields indicated that the energy conversion at larger Tl concentration was highly impaired. Therefore an analysis of the fluorescence images was performed to evaluate whether the areas with high level of non-photochemical quenching overlapped with the discolored leaf regions (Additional file 1: Figure S3). Examples of fluorescence curves averaged over the total leaf area are presented in Additional file 1: Figure S3A. The NPQ parameter for the entire leaf was calculated from these curves and shown in Additional file 1: Figure S3B the NPQ curve has a maximum after the second saturation pulse and later, due to the activation of the photochemical energy dissipation processes resulting in a slow relaxation of NPQ. In leaves with higher concentration of Tl the NPQ parameter reached higher values and the relaxation was slower. The analysis of the NPQ distribution throughout leaf area (Additional file 1: Figure S3C) showed that in the control, H100, and H500 leaves the kinetics of NPQ was typical for non-stressed plants and correlated well with the F_V_/F_M_ and leaf surface morphology (Fig. 4). However, in the discolored leaf samples from plants exposed to 500 and 1,000 μg L^−1^ of Tl the distributions of NPQ were unequal. At 500 μg L^−1^ the NPQ reached a value above 1.5 in the center of leaf where the most evident discoloration occurred (Fig. 4) and the relaxation of NPQ was much longer compared to control leaves. At 1,000 μg L^−1^ of Tl the NPQ reached values close to 2.0 and the relaxation during the actinic light illumination was much slower than that in any other examined sample (Additional file 1: Figure S3C).

### Quantitative and qualitative analysis of chlorophyll and carotenoid content

**Fig. 6:**
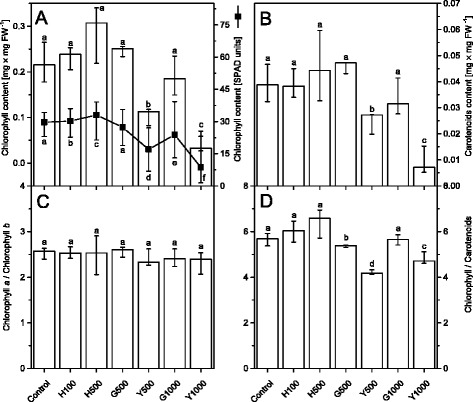
Chlorophyll and carotenoid content in control and Tl-treated *S. alba* leaves. Chlorophyll (**a**) and carotenoid (**b**) content measured after extraction with acetone and the calculated chlorophyll *a* to chlorophyll *b* (**c**) and chlorophylls to carotenoids (**d**) ratios. Additionally the chlorophyll concentration (in relative SPAD values) measured directly from the leaf blade (A, black solid line with full squares). Values are medians from three to six replicates and whiskers show the range of the data; in the case of leaf blade measurements there was at least 20 replicates. Values denoted by the same letter did not differ significantly at *p* = 0.05. Description of samples abbreviation as given in the legend to Fig. 3

It was demonstrated that a diminished chlorophyll (Chl) content and the Chl *a*/*b* ratio under stress conditions indicated degradation of the Chl molecules together with the decomposition of the CP complexes [7, 57]. In our experiments these parameters (Fig. 6) did not change significantly in green leaves of plants exposed to 100 and 500 μg L^−1^ of Tl, suggesting that the CP complexes were not damaged under these conditions. In contrast, in the yellow parts of leaves (Y500, Y1000) the total Chl and carotenoid (Car) content decreased by 50 % and 75 %, respectively in comparison to the control plants. It should be noted that in G1000 samples this effect was also visible but without statistical significance. Furthermore, the Chl *a*/*b* ratio did not change significantly across all plants, while the Chl/Car ratio decreased only in the yellowed parts. These observations confirmed that the degradation of the CP complexes occurred mainly in the yellow parts of leaves while in the green parts the CP complexes remained structurally intact despite higher Tl concentration exposure.

**Fig. 7:**
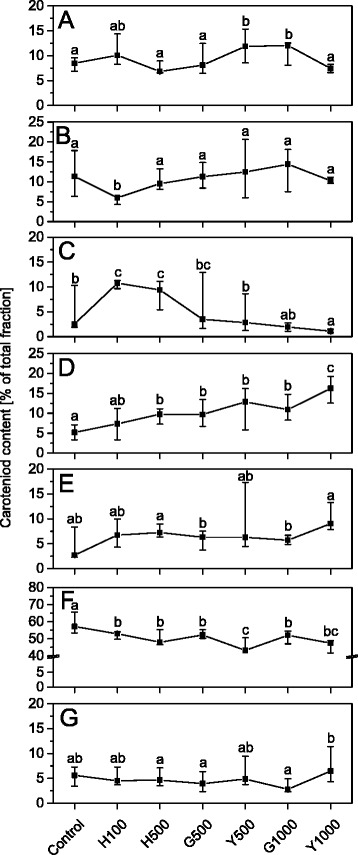
Relative composition of carotenoids in leaves of control and Tl-treated *S. alba* plants. Relative composition of *trans*-neoxanthin – (all-*trans*) (**a**); *cis*-neoxanthin – (9’13), (9’9), (9-*cis*) (**b**); β-carotene (**c**); *trans*- violaxanthin – (all-*trans*) (**d**); *cis*-violaxanthin – (15’-*cis*), (13-*cis*), (9-*cis*) (**e**); *trans*-lutein – (all-*trans*) (**f**)*; cis*-lutein – (9-*cis*), (13’-*cis*) (**g**). Values are expressed as a percentage of all identified and quantified carotenoids and are medians from three to seven replicates and whiskers show the range of the data. Values denoted by the same letter did not differ significantly at *p* = 0.05. Description of samples abbreviation as given in the legend to Fig. 3

The organization of thylakoids and photochemical activity depend on the polar lipid composition, the type of carotenoids associated with the CP complexes [58] and carotenoids directly incorporated into membrane bilayer [59, 60] Therefore, determination of carotenoid composition and speciation is important in assessing the physical properties of thylakoids. In the control plant leaves the lutein in the *trans* conformation reached 56 % of the total carotenoids, while the *cis*- and *trans*-neoxanthin, *cis*- and *trans*-violaxanthin and *cis*-lutein were estimated to be 10 %, 5 % and 5 % of total carotenoids, respectively (Fig. 7). The relative content of other xanthophylls and β-carotene did not exceed 1 % and 5 %, respectively. Traces of zeaxanthin and α-carotene were also found.

The exposure of plants to an increasing Tl concentrations did not significantly affect the molecular forms of neoxanthin (Fig. 7a, b), luteoxanthin (not shown) and antheraxanthin (not shown). The level of the *cis*-lutein did not differ much from the control, but its level increased in Y1000 samples relative to that in G1000 samples (Fig. 7g). Similar variability was observed for the β-carotene content, which increased 2-fold in H100 and G100, but at higher Tl concentration it decreased gradually reaching minimum level in Y1000 samples (Fig. 7c). Nearly linear changes in contents of violaxanthins (Fig. 7d, e) and *trans*-lutein (Fig. 7f) was also observed. The *trans*-violaxanthin level, which in control leaves was about 5 %, increased to 16 % in Y1000 (Fig. 7d), while *trans*-lutein decreased to approximately 45 % in both Y500 and Y1000 samples (Fig. 7f). The *trans*-lutein content in G1000 samples was noticeably higher than those in Y500 and Y1000 samples (Fig. 7f). The analysis of quantitative relationships between different carotenoid species pointed at a significant influence of Tl on the xanthophylls biosynthesis, in particular on the lutein and neoxanthin pathways.

### Analysis of the composition of chlorophyll-protein complexes

**Fig. 8:**
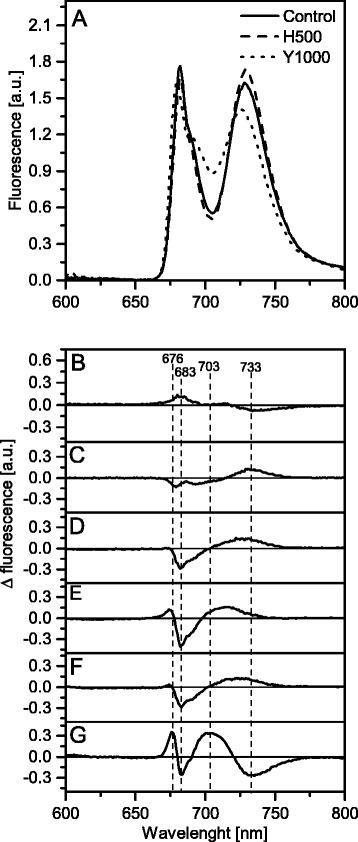
Chlorophyll fluorescence emission spectra (77 K) of *S. alba* thylakoids. Fluorescence was excited at 470 nm for thylakoids isolated from control (solid line), healthy 500 μg L^−1^ of Tl (dashed line) and yellow 1000 μg L^−1^ of Tl (dotted line) leaves (**a**). Fluorescence emission-difference spectra Tl treated-minus-control were calculated for H100 (**b**), H500 (**c**), G500 (**d**), Y500 (**e**), G1000 (**f**), Y1000 (**g**) - description of abbreviation on the Fig. 3 legend. The spectra were normalized to the area of 100 under the spectrum, and the difference spectra (B-G) were calculated for the respective excitation spectra. The presented spectra are representative of three independent experiments

In order to determine the relative contribution of the specific chlorophyll-protein (CP) complexes to the overall fluorescence pattern in thylakoids isolated from plants exposed to different Tl concentrations, the steady-state fluorescence emission spectra at 77 K were measured and normalized to the same area (100) under the spectrum (Fig. 8). The control spectrum of white mustard plant thylakoids (Fig. 8a, solid line) exhibited typical features of the fluorescence emission spectrum of higher plants, that is composed of two bands centered at 682 nm and 729 nm related to the fluorescence from PSII-LHCII and PSI-LHCI, respectively. The PSII-LHCII fluorescence band showed a shoulder around 690 nm which was related to the PSII core complex [6, 61].

In Tl-exposed plants the emission spectrum of thylakoids differed from that in the control, with the ratios of the particular bands varied and the band positions shifted (Fig. 8a dashed and dotted lines). For quantitative determination of the observed changes, the difference between the Tl-exposed and control spectra was calculated (Fig. 8b-g). In H100 leaf samples there were no significant changes in the emission spectra (Fig. 8b). The analysis of leaf samples of plants exposed to 500 μg L^-1^ of Tl (Fig. 8c-e) showed a progressive decrease of the 683 nm band and an increase of the 733 nm band, which was also blue-shifted in the Y500 samples (Fig. 8e). Moreover, in the Y500 samples there was a positive band at 676 nm corresponding to the trimeric LHCII disconnected from PSII [62]. The difference spectra of the G1000 (Fig. 8f) and G500 were very similar but the most pronounced changes were detected in the Y1000 samples (Fig. 8g) where two negative bands at 683 and 733 nm corresponding to a decrease of PSII-LHCII and PSI-LHCI complexes respectively were observed. The positive band at 676 nm was much higher than that in the corresponding Y500 sample. A wide positive band centered at 703 nm corresponding to the aggregated form of LHCII antenna complexes [63] was visible. Summarizing, an increased Tl concentration in leaves appears related to the decrease of the PSII-LHCII and PSI-LHCI supercomplexes and to large increase of the LHCII complexes that are disconnected from the PSII-LHCII, both in the aggregated and trimeric forms.

**Fig. 9:**
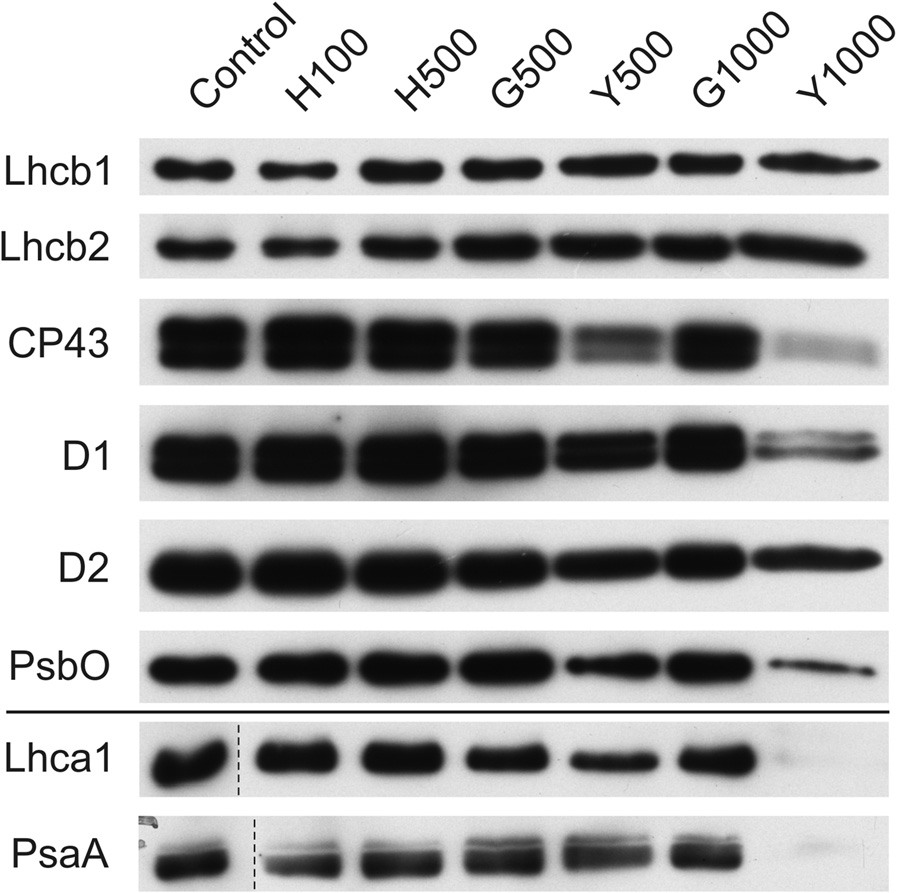
Changes of PSII and PSI antenna and core protein levels. Proteins from control and Tl-treated white mustard leaves were separated by SDS-PAGE followed by immunodetection with antibodies against Lhcb1, Lhcb2, Lhca1 (antenna proteins) and D1, D2, CP43, PsbO, PsaA (core proteins). Samples were loaded on the equal amount of chlorophyll (0.25 μg). Description of samples abbreviation as given in the legend to Fig. 3

The analysis of changes in the protein level (Fig. 9) showed that in the Tl-grown plants, especially in yellow parts of the leaves, the levels of PSII core proteins: D1, D2, CP43 and extrinsic PsbO strongly decreased, which was clearly visible in the Y1000 samples. However, the level of antenna proteins Lhcb1 and Lhcb2 - components of the LHCII complex, remained stable. These observations correlated with the 77 K fluorescence analysis. Additional analysis of the PsaA and Lhca1 proteins - components of the PSI-LHCI supercomplex showed that both antenna (Lhca1) and core (PsaA) protein levels decreased in the Y500 and Y1000 samples in comparison to the controls and in Y1000 they reached undetectable level.

## Discussion

The effect of an increasing dose of Tl on the functional and structural basis of photosynthesis was investigated in this study by many complementary methods. The results allowed to distinguish between different features of Tl toxicity. The overall effect was visible as a decrease in the root tolerance index (Fig. 1), the plant and leaf size, the number of discolored and necrotic leaves (Additional file 1: Figure S1), changes in the mesophyll structure (Additional file 1: Figure S2) and a 50 % decline in the PSI (Y(I)) and PSII (Y(II)) photochemical activities (Fig. 5).

Increased Tl concentration in culture media gradually affected root development and growth. The most characteristic macroscopic change of this effect was a reduction of the root length at highest concentration of the toxicant. However, the tolerance index of plants grown in the lowest applied concentration changed significantly during the experiment – it increased up to the level of control along with the 2-fold decrease of Tl concentration in the medium. Thus, plants were exposed to a decreasing concentration of Tl during the cultivation. This could explain high tolerance of *S. alba* to 100 μg L^-1^ at the end of treatment. For plants treated with 500 μg L^-1^ of Tl, an increase of root tolerance index was also noticed, as well as a decrease of Tl concentration in the medium; however, the concentration of Tl that affected plants during the experiment was still much higher than 100 μg L^-1^. Therefore, the tolerance index of plants treated with 500 μg L^-1^ of Tl in the initial concentration was significantly lower in comparison with plants exposed to 100 μg L^-1^ of Tl at the end of treatment. We assumed that plants exposed to concentration of 500 μg L^-1^ of Tl at the beginning of experiment were moderately affected by toxic effects of thallium. Thus, two types of changes in leaves of these plants were observed. The exposure to 1,000 μg L^-1^ of Tl caused severe effects of toxicity for the whole plant. Root growth was strongly inhibited resulting in a complex reaction of plants by combination of various disturbances, being secondary effects of Tl action (disturbance of water balance, ion homeostasis, and oxidative stress). Therefore, inhibition of photosynthesis in plants exposed to 1,000 μg L^-1^ of Tl was caused by indirect effects of high Tl concentration. Summing up, a gradual effect on the photosynthetic parameters in *S. alba* exposed to increasing concentrations of Tl was due to the transition from lack of toxicity symptoms at 100 μg L^-1^, through moderate toxicity and increasing sensitivity of plants treated with 500 μg L^-1^, to high toxicity and breakdown of the whole plant homeostasis by 1,000 μg L^-1^.

Thallium moves freely across cell membranes and accumulates in cells [24, 64] due to similarity of Tl^+^ and K^+^ ions. The *S. alba* roots accumulate the lowest amounts of Tl because it is effectively transported into the above-ground organs. Considering the biomass, 80 % of the up taken Tl was found in leaves and stems [65]. These data suggest that the leaf cells and the photosynthetic process are important targets of Tl toxicity. This is not different from the action of other heavy metals, which affect directly or indirectly the photosynthetic reactions (see references in Introduction).

Visible signs of excessive heavy metals exposure is chlorosis preceding the necrosis of leaf tissues, advancement of which depends on susceptibility of plant species and metal concentration [66]. The damaged cells are usually clustered in discolored or necrotic spots randomly arranged in the leaf blade in plants treated with Cu, Cd or Zn among others [12, 66–68]. Thallium-induced changes in morphology and photochemical activity gradually spread around the main vascular bundles of leaves (Fig. 4). This effect was most clearly visible in leaves from plants exposed to 500 μg L^−1^ of Tl (Fig. 4d), where the overall moderate toxicity (Fig. 1) was analyzed in three selected leaf areas. The concentration of Tl in Y500 was twice as high as in H500 and G500 samples. Noticeable concentration of Tl(III) was detected in H500 and G500 leaves (Fig. 2), despite the fact that the nutrient solutions contained Tl(I) only. Oxidation of Tl(I) took place in plant tissue and the content of Tl(III) reached up to 10 % of total Tl, as we showed in our previous study on mustard plants grown in spiked soil [69]. In comparison to Tl(III) the monovalent thallium is thermodynamically more stable and less reactive which makes it the dominant form of Tl. In contrast to Tl(I) the trivalent thallium forms very stable complexes with many ligands thus resulting with reduced toxicity [70].

As was establish in model systems and in animal cells the reactions of Tl(I) with reduced compounds produce Tl(0) and organic free radicals [71]. In plant cells these reactions might proceed by direct interactions with reduced components of the photosynthetic chain. Tl(0) and organic radicals subsequently react with the molecular oxygen producing superoxide (O_2_^-•^), which is later dismutased to other reactive oxygen species (ROS) both by enzymatic and autocatalytic reactions [71]. ROS initiate the peroxidative chain reactions [40, 72, 73], among others they react with Tl(I) producing thallic ions Tl(III), a dangerous strong oxidant (E_0_ Tl^3+^/Tl^+^ = 1.2 V). Tl(III) can initiate the lipid or protein peroxidation and cause regeneration of the more reduced Tl(I), which can react again with ROS [72, 74]. This hypothetical Tl-ROS cycle takes place most likely in chloroplasts in thylakoid membranes, but further studies are required for elucidation of the molecular mechanism.

Accumulation of Tl(III) was only observed in H500 and G500 samples (Fig. 2) and in these samples the photochemical yield Y(I) and Y(II) decreased by 20 % of the control levels (Fig. 5) without changes in the maximal quantum yield of PSII (F_V_/F_M_) (Fig. 4). The chlorophyll and carotenoid contents (Fig. 6) as well as arrangement of the thylakoid membranes (Fig. 3) remained unchanged and in G1000 samples these parameters resembled those observed in H500 and G500 samples.

The maximal quantum yield (F_V_/F_M_) is related to open reaction centers and decreases in impaired PSII complexes [75–77], whereas the Y(I) and Y(II) depend not only on the connection between reaction centers but also on the amount of down-regulation processes [78]. Simultaneously with the decrease of the photochemical activity in the H500 and G500 samples an increase of the non-photochemical parameters was observed (Fig. 5), which indicates a limitation in the oxidation (PSI) and reduction (PSI, PSII) of photosystems [78]. Therefore we suggest that thallium influences the photosynthesis without degradation of the CP complexes and grana structure in the H500, G500 and partly in G1000 samples. Noticeably the percentage level of Tl(III) in the total amount of thallium suggests that the Tl-ROS cycle is restricted probably by antioxidant systems and only a direct interaction of Tl(I) with reduced electron carriers of the photosynthetic chain occurs. Furthermore, in H500 samples the increase of both F_0_ and F_M_ values due to a significant increase of chlorophyll concentration (Figs. 4 and 6) can be interpreted as an attempt to acclimatization of plants to a higher Tl concentration.

The analysis of the protein composition of the main thylakoids complexes in H500, G500 and even in G1000 samples (Fig. 9) did not show changes in protein levels nor in the ratios of core and antennae proteins. These data taken together with a stable value of the maximal quantum yield of PSII (Fig. 4) suggested the integrity of the CP complexes was not affected. However, low-temperature fluorescence spectra (Fig. 8) indicated a decrease in the connectivity between the external antennae and core complexes of PSII with a simultaneous increase of the PSI fluorescence intensity. This observation can be related to a certain energy spillover from PSII to PSI [79, 80]. The basic unit of LHCII-PSII consists of dimeric form of PSII, two copies of monomeric antennae and two LHCII trimers. Moreover, up to four other LHCII trimers might be weakly associated with PSII dimer [5, 81]. The LHCI-PSI complex in unstacked regions of membranes might bound the LHCII trimer, creating a new supercomplex [82]. It appears probable that in H500, G500 and G1000 samples the part of loosely associated LHCII trimers is bound to the LHCI-PSI complexes without visible changes in the grana structure (Fig. 3). Despite that in H500 and G500 samples the overall carotenoid levels did not decrease (Fig. 6), noticeable qualitative changes of the carotenoid species were observed (Fig. 7). Variations of the relative amount of violaxanthin, lutein and carotene might modulate the membrane rigidity and support the protein diffusion [59, 83].

Thus, we have shown that the first non-destructive stage of the thallium toxicity is associated with two overlapping effects: (*i*) direct influence of Tl on photochemistry and (*ii*) partial rearrangements of the CP complexes. Furthermore, the early phase of toxicity in young H500 leaves was associated with an active response to the Tl stress by an increase of the chlorophyll and carotene concentration (Figs. 6 and 7).

The next stage of thallium toxicity, probably more closely related to an indirect toxic effect (Fig. 1), revealed by the simultaneous functional disorder of PSI and PSII photochemistry (Fig. 5) and the PSII quantum yield (Fig. 4), appears associated with the CP complexes degradation [84]. In Y500 and Y1000 samples a substantial decrease of the chlorophyll and carotenoid levels (Fig. 6) was associated with a 2- and 3-fold increase of Tl(I) concentration, respectively, but thallium oxidation to Tl(III) was not observed (Fig. 2). This might suggest an increase of the Tl-ROS cycle rate and insufficient activity of the antioxidant systems, which may lead to oxidation of the thylakoids pigments [40]. Large deficiency of the β-carotene in Y1000 samples (Fig. 7c) appears indicative of these processes.

Since F_0_ originated from the chlorophylls associated with antennae complexes almost triple increase of F_0_ fluorescence in Y500 and Y1000 samples (Fig. 4) indicated a decreased energy transfer from the LHCII to PSII cores due to disconnection of the antennae from LHCII-PSII [75, 78]. Furthermore, the appearance of 676 and 700 nm bands in the 77 K difference spectra (Fig. 8e, g) attributed to the emission from the trimeric or aggregated form of the LHCII non-associated with PSII reaction centers [6, 52, 62, 79] confirmed that the increase of F_0_ is related to disintegration of the LHCII-PSII supercomplexes [75]. Moreover, higher values and slow relaxation of the NPQ parameter (Additional file 1: Figure S3) observed in Y500 and Y1000 leaves is usually related to the aggregation of LHCII [85, 86]. A noticeable decrease in the relative amount of the PSII core proteins (D1, D2, CP43) without significant changes in the level of the LHCII proteins (Lhcb1, Lhcb2) lead to an increase of the antennae/core protein ratio (Fig. 9) and suggests rearrangements or a partial decline of the LHCII-PSII supercomplexes. A significant decrease in the PsbO protein level (Fig. 9) may cause dissociation of the Mn-cluster, inhibition of the oxygen evolution and a decrease of the PSII quantum yield (Fig. 4) [11, 75]. It is worth noticing that the levels of the core (PsaA) and antennae (Lhca1) proteins of the LHCI-PSI complex were stable in all samples but were absent in the Y1000 parts of leaves (Fig. 9). The LHCII protein levels in Y500 and Y1000 samples were the same as in the other part of leaves, while the level of the core proteins of PSII was lower but these proteins were still present (Fig. 7). The structure of grana stacks is mainly stabilized by an ordered arrangement of the LHCII-PSII and LHCII complexes [5], while the unstacking of grana is related to the disconnection of LHCII from supercomplexes and random distribution of the photosystems in the lateral plane of the thylakoid membrane [79]. Therefore, we propose that the decrease in the content and disordered arrangements of supercomplexes, mainly LHCII-PSII, were responsible for the decline of the grana structure observed in Y500 and Y1000 samples (Fig. 3).

## Conclusions

In this study we have identified two phases of thallium toxicity. In the first phase a direct influence of thallium on the photochemistry reactions and partial rearrangements of the photosynthetic complexes was observed without significant changes in the pigment and protein levels, and in the chloroplast structure. The second phase, probably indirect destructive phase of thallium toxicity that was observed in the discolored leaf areas only, is associated with massive oxidation of pigments, decrease of the photosynthetic core protein levels, disorder of the CP complexes and grana disappearance. The border between the first (non-destructive) and second (destructive) phase of thallium toxicity is not very distinct as the two phases overlapped. The intensity of thallium toxicity is proportional to the Tl migration outside the vascular bundles, its accumulation in tissues, and time of action. The toxicity seems to be related to the ratio between the two observed chemical forms of thallium: Tl(I) and Tl(III).

## Additional file

Additional file 1: Figure S1. Morphology of white mustard plants grown in the control conditions (**A**) and in the presence of thallium in concentration 100 μg L^−1^ (**B**), 500 μg L^−1^ (**C**) and 1,000 μg L^−1^ (**D**). Bar = 2.5 cm. **Figure S2.** Changes of leaf blade architecture of white mustard plants grown in the presence of thallium revealed by bright field microscopy. Pictures show leaf blade cross-sections from control (**A**) and Tl-treated plants (**B-G**): 100 μg L^−1^ (H100) (**B**); 500 μg L^−1^ healthy green leaves, which showed no changes in morphology comparing to the control cultivation (H500) (**C**), green (G500) (**D**) and yellow (Y500) (**E**) parts of the affected leaves; 1,000 μg L^−1^ green (G1000) (**F**) and yellow (Y1000) (**G**) parts of the affected leaves. **Figure S3.** Analysis of non-photochemical quenching parameter (NPQ) in the dark adapted white mustard leaves from control and Tl-treated plants presented on Fig. 3. (**A**) an exemplary fluorescence traces for control, 500 μg L^−1^ of Tl and 1,000 μg L^−1^ of Tl leaves recorded during exposition of dark-adapted leaves to actinic light illumination. (**B**) Changes of NPQ parameter calculated for the entire leaf area on the basis of recorded fluorescence traces during actinic light illumination. (**C**) Visualization of the induction and relaxation of NPQ throughout the leaf. The images are representative for at least ten leaves from each treatment. Description of samples abbreviation as given in the legend to Fig. 3. (PDF 629 kb)

## Abbreviations

Car: Carotenoids
Chl: Chlorophyll
CP: Chlorophyll-protein
DTPA: Diethylenetriaminepentaacetic acid
F_0_: Minimal fluorescence level
F_M_: Maximal fluorescence level
F_V_/F_M_: Maximal quantum efficiency of photosystem II
LHCI/II: Light harvesting complex associated with PSI and PSII respectively
NPQ: Non-photochemical quenching
OEC: Oxygen evolving complex
PAR: Photosynthetically active radiation
P700: Reaction center of photosystem I
PSI: Photosystem I
PSII: Photosystem II
ROS: Reactive oxygen species
Y(I): Photochemical quantum yield of PSI
Y(II): Effective PSII quantum yield
Y(NA): Nonphotochemical quantum yield of PSI – acceptor side limitation
Y(ND): nonphotochemical quantum yield of PSI – donor side limitation
Y(NO): PSII quantum yield of nonregulated energy dissipation
Y(NPQ): PSII quantum yield of regulated energy dissipation
