# Supplementary material for: Overlapping toxic effect of long term thallium exposure on white mustard (Sinapis alba L.) photosynthetic activity
Source: BMC Plant Biol. 2016 Sep 2;16:191. doi: 10.1186/s12870-016-0883-4 (PMC5009500; doi:10.1186/s12870-016-0883-4)
Supplement: Additional file 1: Figure S1. — Morphology of white mustard plants grown in the control conditions (A) and in the presence of thallium in concentration 100 μg L−1 (B), 500 μg L−1 (C) and 1,000 μg L−1 (D). Bar = 2.5 cm. Figure S2. Changes of leaf blade architecture of white mustard plants grown in the presence of thallium revealed by bright field microscopy. Pictures show leaf blade cross-sections from control (A) and Tl-treated plants (B-G): 100 μg L−1 (H100) (B); 500 μg L−1 healthy green leaves, which showed no changes in morphology comparing to the control cultivation (H500) (C), green (G500) (D) and yellow (Y500) (E) parts of the affected leaves; 1,000 μg L−1 green (G1000) (F) and yellow (Y1000) (G) parts of the affected leaves. Figure S3. Analysis of non-photochemical quenching parameter (NPQ) in the dark adapted white mustard leaves from control and Tl-treated plants presented on Fig. 3. (A) an exemplary fluorescence traces for control, 500 μg L−1 of Tl and 1,000 μg L−1 of Tl leaves recorded during exposition of dark-adapted leaves to actinic light illumination. (B) Changes of NPQ parameter calculated for the entire leaf area on the basis of recorded fluorescence traces during actinic light illumination. (C) Visualization of the induction and relaxation of NPQ throughout the leaf. The images are representative for at least ten leaves from each treatment. Description of samples abbreviation as given in the legend to Fig. 3. (PDF 629 kb) [file 12870_2016_883_MOESM1_ESM.pdf]

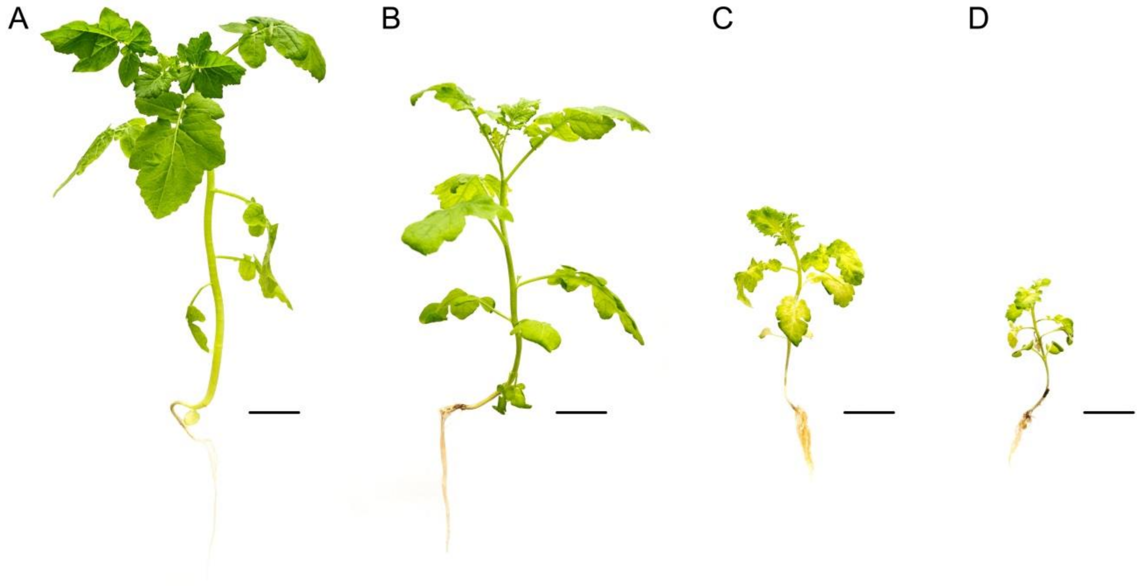

Figure S1. Morphology of white mustard plants grown in the control conditions (A) and in the presence of thallium in concentration 100 μg L^{-1} (B), 500 μg L^{-1} (C) and 1000 μg L^{-1} (D). Bar = 2.5 cm.

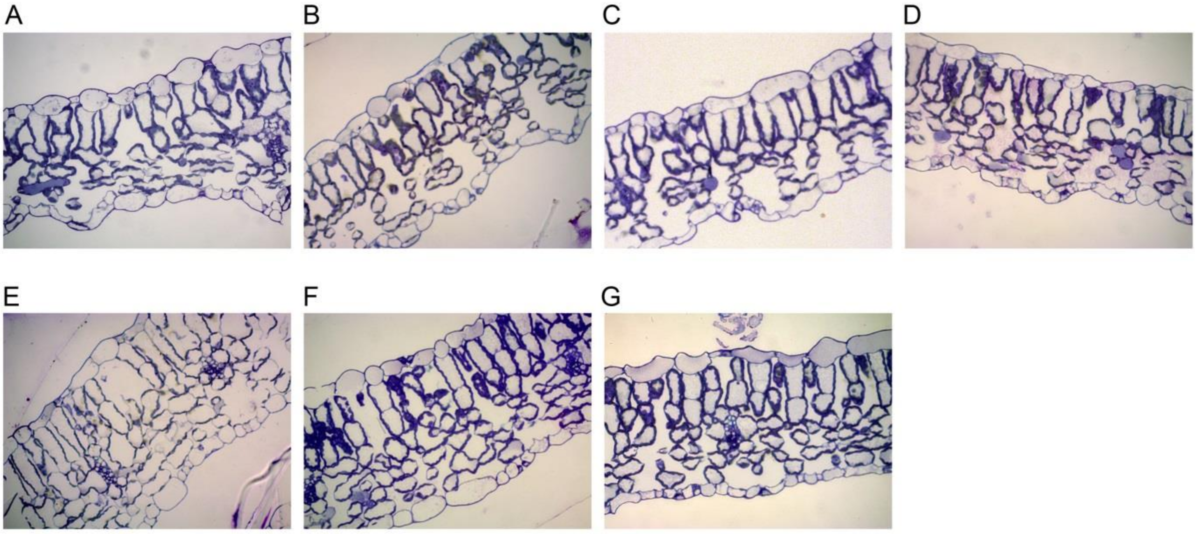

Figure S2. Changes of leaf blade architecture of white mustard plants grown in the presence of thallium revealed by bright field microscopy. Pictures show leaf blade cross-sections from control (A) and Tl-treated plants (B-G): 100 μg L^{-1} (H100) (B); 500 μg L^{-1} healthy green leaves, which showed no changes in morphology comparing to the control cultivation (H500) (C), green (G500) (D) and yellow (Y500) (E) parts of the affected leaves; 1000 μg L^{-1} green (G1000) (F) and yellow (Y1000) (G) parts of the affected leaves.

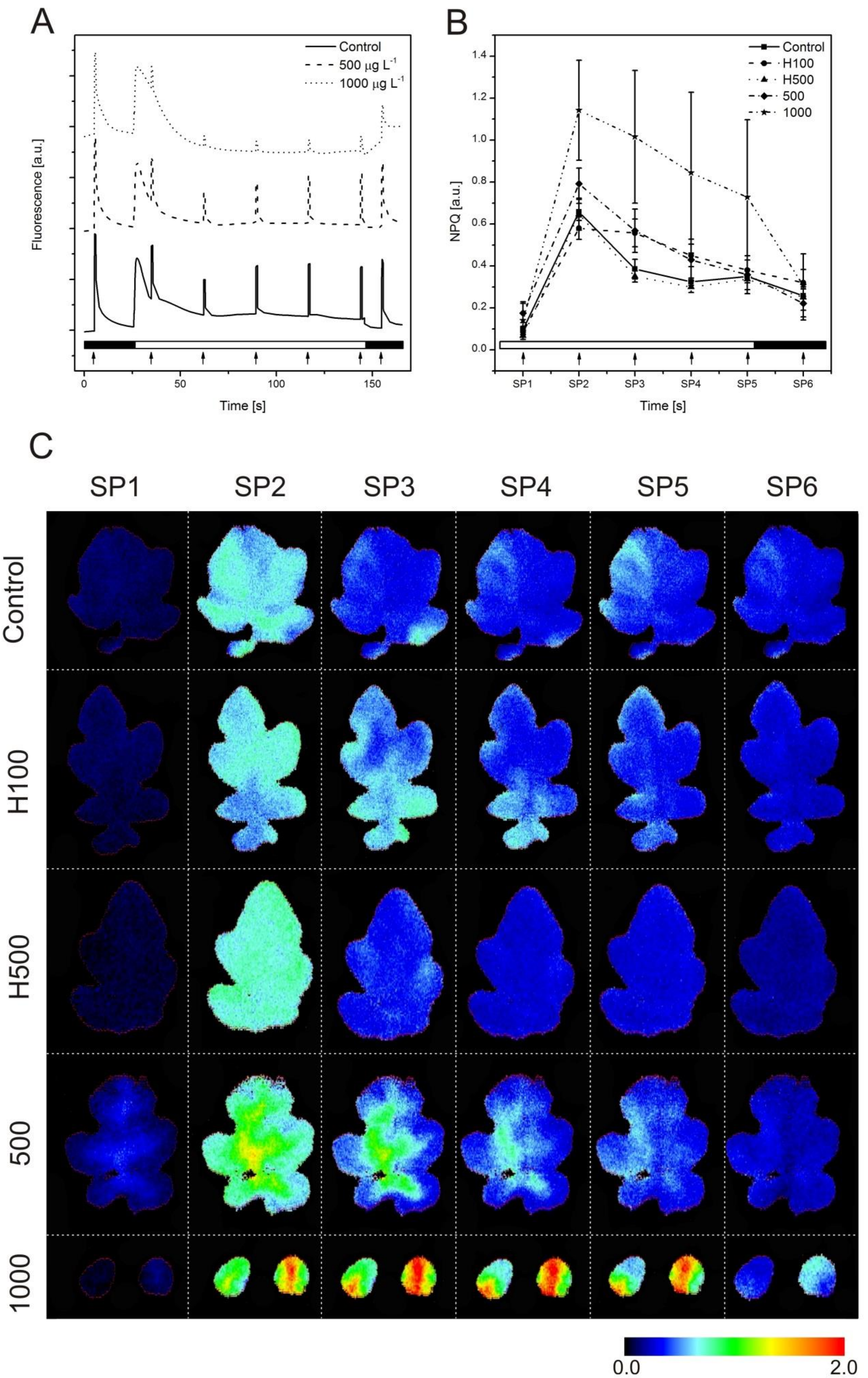
A
Control
500 μg L⁻¹
1000 μg L⁻¹
Fluorescence [a.u.]
0
50
100
150
Time [s]
B
Control
H100
H500
500
1000
NPQ [a.u.]
0.0
0.2
0.4
0.6
0.8
1.0
1.2
1.4
SP1
SP2
SP3
SP4
SP5
SP6
Time [s]
C
SP1
SP2
SP3
SP4
SP5
SP6
Control
H100
H500
500
1000
0.0
2.0

Figure S3. Analysis of nonphotochemical quenching parameter (NPQ) in dark adapted white mustard leaves from control and Tl treated plants presented on Fig. 3. (A) an exemplary fluorescence traces for control, 500 μg L^{-1} Tl and 1000 μg L^{-1} Tl leaves recorded during exposition of dark adapted leaves to actinic light illumination. (B) changes of NPQ parameter calculated for the entire leaf area on the basis of recorded fluorescence traces during actinic light illumination. (C) visualization of the induction and relaxation of NPQ throughout the leaf. The images are representative for at least ten leaves from each treatment. Description of the samples is the same as on Fig. 3.
